# Ochratoxin A biodegradation by *Agaricus campestris* and statistical optimization of cultural variables

**DOI:** 10.1007/s10068-023-01417-8

**Published:** 2023-09-14

**Authors:** Tuncay Söylemez, Mustafa Yamaç, Ayşe Betül Eninanç, Zeki Yıldız

**Affiliations:** 1 Institut Für Lebensmittelchemie, Gottfried Wilhelm Leibniz Universtät Hannover, Callinstraβe 5, 30167 Hannover, Germany; 2 Department of Biology, Faculty of Science and Letters, Eskisehir Osmangazi University, 26040 Eskisehir, Turkey; 3 Department of Statistics, Faculty of Science and Letters, Eskisehir Osmangazi University, 26040 Eskisehir, Turkey

**Keywords:** Ochratoxin A, Biodegradation, *Agaricus campestris*, Statistical optimization, Plackett–Burman design, Box–Behnken methodology

## Abstract

The goal of this study is to identify the optimum conditions for ochratoxin A (OTA) biodegradation by the supernatant of *Agaricus campestris* strain. The Plackett–Burman and Box–Behnken methods were used to determine optimum OTA degradation conditions of *Agaricus campestris* under various incubation conditions. The Plackett–Burman method was planned through 16 varied experiments with 15 variants. The three most potent variants, sucrose, yeast extract and wheat bran, were selected using the Box–Behnken methodology. Ochratoxin A biodegradation ratio of 46.67% has been specified in only 1 h under ideal growing conditions. This is the first report on the optimization of OTA biodegradation by *Agaricus campestris*. When compared to previously published articles, it can be asserted that *Agaricus campestris* has promise based on its OTA biodegradation ratio in only 1 h of reaction time.

## Introduction

**Fig. 1:**
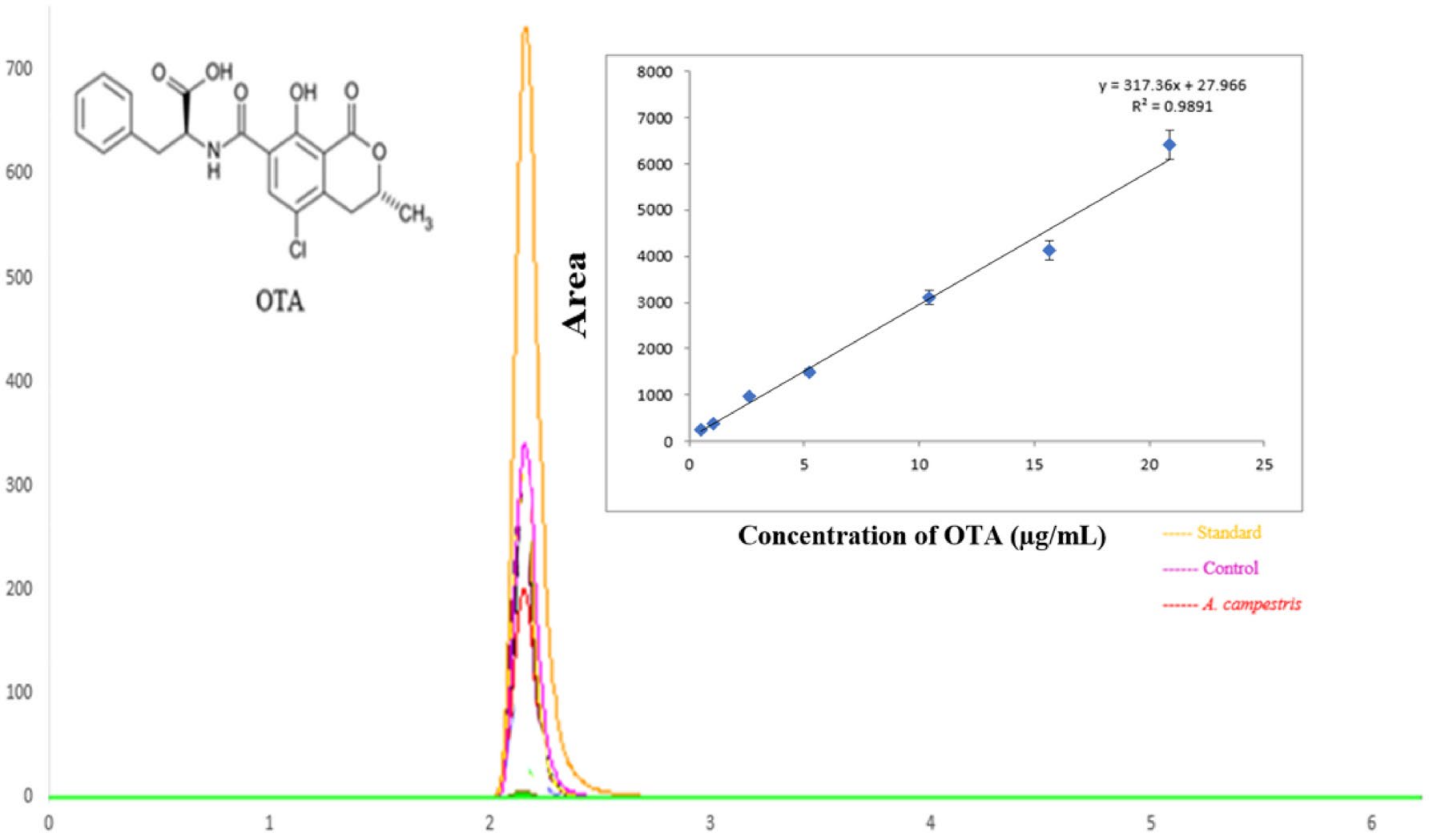
Chromatogram and standard curve of OTA in HPLC analysis

Ochratoxins constitute the largest mycotoxin group first identified after the discovery of aflatoxins. Ochratoxin A (OTA), (C_20_H_18_ClNO_6_; molecular weight: 403.8) is a white, odorless, heat-stable, crystalline solid agent (melting point: 168–173 °C) with poor aqueous solubility and is the most toxic member in the group of ochratoxins (Bragulat et al., 2001). It has been determined that OTA is produced by approximately 21 species, including *Aspergillus, Neopetromyces,* and *Penicillium* species (Wang et al., 2016). Biosynthetically, ochratoxins are pentaketides, consisting of dihydroisocoumarin coupled to 8-phenylalanine. Only OTA and very rarely its dechloroanalogue, ochratoxin B (OTB), are found in foods and feed (Fig. 1). However, 4-hydroxy derivatives such as methyl and/or ethyl esters, as well as the ochratoxin-isocoumarin core of OTB, have been found in mold culture filtrates (Pohland, 1992).

Ochratoxin can competitively inhibit phenylalanine tRNA synthesis and block protein formation. Moreover, it indirectly causes oxidative DNA damage by DNA fusion (Loi et al., 2017). Potential nephrotoxic, teratogenic, genotoxic, and carcinogenic properties of OTA have been emphasized (Petchkongkaew et al., 2008). A role in the aetiology of Balkan endemic nephropathy and its association with urinary tract tumors has also been proven. OTA is a reason for Balkan endemic nephropathy, and this disease is associated with urinary tract tumors (el Khoury and Atoui, 2010). The carcinogenic potential of OTA was reported as a whole monograph by the International Agency for Research on Cancer (IARC) in 1993 (IARC, 1993).

Varieties of commodities are contaminated with OTA, and relatively high contamination levels are found in corn, rye, and coffee (Joint FAO/WHO Expert Committee on Food Additives, 2001). The maximum limits for OTA in a wide range of foodstuffs have been established by European Union Commission Regulation (EC) No 1881/2006, which has limited OTA to 0.5–10 µg/kg in different foodstuffs like infant formula and dried fruits (EC, 2016; https://eur-lex.europa.eu/legal-content/EN/ALL/?uri=CELEX%3A32006R1881). Provisions for methods of sampling and analysis for the official control of OTA are also laid down in Commission Regulation (EC) No. 401/2006.

A study of over 70,000 individual analyses of 19,000 feed and feed production goods samples from various countries found that hazardous mycotoxin, including OTA, contaminated 72% of the samples. In addition, 38% of the samples were co-contaminated with two or more mycotoxins. Although the mycotoxin concentration was at an acceptable level according to European Union Commission Regulation (EC) No 1881/2006, in most cases, synergistic interactions of mycotoxins might have adverse effects (Schatzmayr and Streit, 2013).

When evaluating the potential economic impacts of the recently proposed OTA, using the maximum limits for foodstuffs set by Health Canada as an example, the estimated annual losses to Canadian food producers could exceed 260 million Canadian dollars. In 2006, the owner of a grain mill was accused and arrested of importing 58,000 tons of Canadian wheat contaminated with 15 µg/kg OTA (Bayman and Baker, 2006). Therefore, it can be argued that OTA contamination, except for remarkable health risks, can cause serious economic losses in agriculture depending on the rejection of imported food and feed.

Researchers have been studying mycotoxin degradation and decontamination in feed and food for several decades. There are many approaches, including physical, chemical, and biological principles. In addition, some researchers have used a combination of these approaches to remove or degrade mycotoxins without affecting the quality of food and feed (Muhialdin et al., 2020). A lot of chemicals have shown potential to remove some of the mycotoxin contamination in foods. Some of these chemicals are calcium hydroxide, monomethylamine, ammonia, sodium hydroxide, calcium hydroxide, ozone, and chlorine (Karlovsky et al., 2016). When they destroy the mycotoxin, they also destroy some of the valuable nutrients. Physical methods such as dry cleaning, milling, color sorting, irradiation, floating, washing with water, and removal of damaged grains have limited effect on the removal of mycotoxins. In practice, using physical and chemical methods is limited due to problems such as safety issues, nutritional value loss, insensitivity, high cost, and incomplete detoxification. (Verheecke et al., 2016). As the most hopeful alternative, the biological degradation mechanism has some advantages over the other degradation methods, such as mild reaction conditions, stability in food quality, high detoxification efficiency, and stableness. (Samuel et al., 2014). Microbial detoxification is defined as the biotransformation of mycotoxins by enzymes and their metabolites produced by microorganisms into less- or non-toxic compounds (Deshpande, 2002). Biological agents and their enzymes often perform biotransformation with high degradation success, are environmentally friendly, and cause minimal damage to food (Boudergue et al., 2009).

A great number of microorganisms efficient in adsorbing, degrading, and detoxifying OTA are announced in the literature, and some experimental processes have been developed. There are two pathways for microbial degradation in OTA biodegradation. To begin with, the OTA can be biodegraded by hydrolyzing the amide bond that connects the L-phenylalanine molecule to the OTα component. Another pathway is that OTA can be degraded through the hydrolysis of the lactone ring (Karlovsky, 2016). Ruminants are able to biodegrade OTA following the pathway that yields phenylalanine and OTα, with rumen protozoa being primarily responsible (Kiessling et al., 1984). The microbial degradation of OTA was first reported by the aerobic bacterium *Phenylobacterium immobile* (Wegst and Lingens, 1983). The following bacteria have been published in the literature that are capable of OTA biodegradation, detoxification, and adsorption: *Phenylobacterium immobile*, *Acinetobacter calcoaceticus*, *Bacillus licheniformis*, *B. subtilis*, *Bifidobacterium bifidum*, *Lactobacillus delbrueckii* subsp. *bulgaricus*, *L. helveticus*, *L. acidophillus*, (Abrunhosa et al., 2010).

Although OTA biodegradation by *Saccharomyces cerevisiae* and other yeast was informed, the obtained results could be wrong because of wall adsorption mechanism (Abrunhosa et al., 2010). On the other hand, there are some studies that prove OTA biodegradation by yeasts. For example, *Trichosporon* spp., *Rhodotorula* spp., and *Cryptococcus* spp. gave some good results with their ability to biodegrade OTA through the cleavage of the amide bond and release OTα (Schatzmayr et al., 2013). Some filamentous fungi can biodegradation of OTA. The OTA biodegradation capacity of microfungi described in the literature such as *Aspergillus niger*, *A. fumigatus*, *A. japonicus*, *A. carbonarius*, *Botrytis cinerea*, *Rhizopus homothallicus*, *R. oryzae*, *R. stolonifer* and other *Rhizopus* species (Abrunhosa et al., 2010; Varga et al., 2005). Even if two macrofungi species, *Phanerochaete chrysosporium* and *Pleurotus ostreatus*, are reported for OTA biodegradation (Engelhardt, 2002), there is no report for OTA degradation by *Agaricus campestris*, an edible mushroom. In the present study, OTA biodegradation by *Agaricus campestris* and statistical optimization by using Plackett–Burman and Box–Behnken methodologies were reported for the first time.

## Material and methods

### Chemicals and media

Ochratoxin A (purity > 97%) was acquired from Sigma-Aldrich (Taufkirchen, Germany). All chemicals and medium components were procured from Merck (Darmstadt, Germany).

### Biodegradation agent

The fungal culture studied in this study (OBCC 5048) was isolated from a fruiting body sample that was collected from Ayva village, Mustafakemalpaşa, Bursa, Turkey. The isolate was chosen among 94 macrofungal strains for OTA biodegradation in a screening study conducted by our research group very recently (Söylemez and Yamaç, 2021). Fruiting bodies from the isolated sample were ascertained depending on conventional morphological macro- and micro-characters referred to in the appropriate literature (Moser, 1983).

To verify the conventional identification of the species with molecular methods, the internal transcribed spacer (ITS) rDNA region sequence of the isolate was determined and compared with alignments in the GenBank database (http://www.ncbi.nlm.nih.gov/genbank/). For that purpose, the OBCC 5048 isolate was grown on potato malt peptone (PMP) medium (potato dextrose broth 24.0 g/L, malt extract 10.0 g/L, and peptone 1.0 g/L). The mycelial biomass was harvested, washed with sterile distilled water (SDW), transferred into Eppendorf tubes, and lyophilized. DNA was isolated from the lyophilized mycelia using the modified CTAB method (Murray and Thompson, 1980). Then, PCR amplification of the rDNA region was performed in a 50-μL reaction mixture using ITS1 (5′-TCCGTAGGTGAACCTGCGG-3′) and ITS4 (5′-TCCTCCGCTTATTGATATGC-3′) primers (White et al., 1990). The PCR program was as follows: 4 min at 94 °C for 1 cycle; 30 s at 94 °C, 55 s at 56 °C, 30 s at 72 °C for 35 cycles; 10 min at 72 °C for 1 cycle (Applied Biosystems, Verity). The PCR product was purified and then sequenced by the Life Sciences Research and Application Center, Gazi University, Turkey. The obtained rDNA region sequence was used to conduct a BLAST search in the GenBank database.

The pure culture from the isolate was kept on a Potato Dextrose Agar (PDA) slant at 4 °C and regularly refreshed, then transferred to new PDA plates to inoculate an actively growing strain for the study (Söylemez et al., 2020).

### Inoculum preparation

*A. campestris* OBCC 5048 was grown on a PDA. Five mycelial discs (6 mm diam.) were cut from the edge of the colony. Discs were transferred to 100 mL of PMP for inoculation. The preculture conditions in the medium are 28 °C at 100 rpm for 4 days. Pellets were harvested and rinsed out three times with sterile distilled water (SDW), and the inoculant was prepared via homogenization of the cell suspension for 20 s at 1-min intervals with a Waring laboratory blender (Heidolph Silent Crusher M, Germany). In all experimental groups, this mycelium suspension was used as an inoculant (Söylemez et al., 2020).

### Submerged fermentation

The Modified Kirk Medium (g/L; glucose 10, soytone 5, yeast extract 1, wheat bran 0.2, ammonium tartrate 2, CaCl_2_⋅H_2_O 0.1, MgSO_4_⋅7H_2_O 0.5, KH_2_PO_4_ 2, trace element solution 1, pH 5.00) was used as medium in submerged fermentation. *A. campestris* OBCC 5048 was grown for 10 days in darkness at 125 rpm and 28 °C. When the incubation period was completed, cultures were harvested and filtrated (0.45 µm). Culture fermentation filtrates obtained from *A. campestris* were used as an enzyme source for OTA biodegradation (Söylemez et al., 2020).

### Experimental design for data analysis

Optimization studies were conducted to increase OTA biodegradation by *A. campestris* with submerged fermentation. Therefore, the nutritional and environmental factors of the culture were studied in two consecutive steps, using the Plackett–Burman design (PB) and Box–Behnken (BB) methodology, respectively.

#### Screening of important factors using the Plackett–Burman design

The Plackett–Burman statistical design (Plackett and Burman, 1946) was used as a first step in the optimization study to determine effective variants. Plackett–Burman is a first-order polynomial model used to screen plenty of independent variations and recommends fewer variables for a higher optimization fraction in a two-level factorial design. It also enables the investigation of “n − 1” variables with a minimum “n” experiment. The basis effect was calculated for all factors via the difference between the average of measurements observed at the high setting (+ 1) and the average of measurements at the low setting (− 1). The most important medium components or incubation conditions were selected to improve OTA biodegradation by using this statistical design. To select the best medium components, the Kirk medium was modified. In this study, 10 medium components and 5 environmental factors (totaling fifteen variables) were screened in 16 combinations (Table 1).

**Table 1:** Plackett–Burman design matrix for evaluating variables for OTA degradation and observed degradation ratios by *Agaricus campestris*

Exp. No	Variables															OTA Degradation (%)
	X_**1**_	X_**2**_	X_**3**_	X_**4**_	X_**5**_	X_**6**_	X_**7**_	X_**8**_	X_**9**_	X_**10**_	X_**11**_	X_**12**_	X_**13**_	X_**14**_	X_**15**_	
1	2	2	2	2	0	0.2	0	0.2	0.2	0	25	6	100	2.5	8	19.12
2	2	2	2	0	2	0	0.2	0.2	0	0	30	4	100	2.5	12	37.99
3	2	2	0	2	0	0.2	0.2	0	0	0.2	25	4	100	7.5	12	47.79
4	2	0	2	0	2	0.2	0	0	0.2	0	25	4	150	7.5	12	42.16
5	0	2	0	2	2	0	0	0.2	0	0	25	6	150	7.5	12	37.99
6	2	0	2	2	0	0	0.2	0	0	0	30	6	150	7.5	8	14.46
7	0	2	2	0	0	0.2	0	0	0	0.2	30	6	150	2.5	12	43.87
8	2	2	0	0	2	0	0	0	0.2	0.2	30	6	100	7.5	8	19.12
9	2	0	0	2	0	0	0	0.2	0.2	0.2	30	4	150	2.5	12	39.46
10	0	0	2	0	0	0	0.2	0.2	0.2	0.2	25	6	100	7.5	12	43.63
11	0	2	0	0	0	0.2	0.2	0.2	0.2	0	30	4	150	7.5	8	13.97
12	2	0	0	0	2	0.2	0.2	0.2	0	0.2	25	6	150	2.5	8	18.38
13	0	0	0	2	2	0.2	0.2	0	0.2	0	30	6	100	2.5	12	36.52
14	0	0	2	2	2	0.2	0	0.2	0	0.2	30	4	100	7.5	8	23.77
15	0	2	2	2	2	0	0.2	0	0.2	0.2	25	4	150	2.5	8	22.06
16	0	0	0	0	0	0	0	0	0	0	25	4	100	2.5	8	4.90

X_1_–X_5_ Carbon source % (X_1_, Glucose; X_2_, Fructose; X_3_, Sucrose; X_4_, Lactose; X_5_, Starch); X_6_–X_9_ Nitrogen source % (X_6_, Yeast extract; X_7_, Meat extract; X_8_, Soytone; X_9_, Casein); X_10_, Wheat bran %; X_11_, Incubation temperature °C; X_12_, pH; X_13_, Agitation rpm; X_14_, Inoculum amount %v/v; X_15_, Incubation time day

#### Optimization of significant variables using Box–Behnken methodology

Wheat bran, sucrose, and yeast extract were figured out to be efficient factors for OTA biodegradation by using the PB design. The response surface method (RSM) was inquired using the Box–Behnken methodology to ascertain the optimum concentrations of these medium components as a second step of optimization. Totally, 17 fermentation groups were used experimentally to determine the singular and interactive effects of all medium components on OTA biodegradation. The Box–Behnken methodology used to determine the levels of these independent variables is included in Table 2. Seventeen tests at low (− 1), medium (0), and high (+ 1) levels of every three factors were set up as an experimental design. The behavioral system was explained by the following quadratic equation:

1 $$Y = \beta_{0} + \sum {\beta_{i} } x_{i} + \sum {\beta_{ij} } x_{i} x_{j} + \sum {\beta_{ii} } x_{i}^{2}$$

where yield (Y): The predicted response, β_0_: The intercept term, β_i_: Linear effect, β_ii_: The squared effect, β_ij_: The interaction effect, x_i_ and x_j_: The encoded independent variables.

**Table 2:** The predicted and observed OTA degradation results for Box–Benken Design matrix

Exp. no	Variables			OTA Degradation (%)	
	X_3_ (Sucrose; %)	X_6_ (Yeast Extract; %)	X_10_ (Wheat Bran;%)	Observed	Predicted
1	2 (0)	0.3 (+ 1)	0.3 (+ 1)	46.22	45.78
2	2 (0)	0.2 (0)	0.2 (0)	40.67	41.29
3	1 (− 1)	0.2 (0)	0.1 (− 1)	40.89	39.55
4	2 (0)	0.2 (0)	0.2 (0)	41.78	41.29
5	1 (− 1)	0.1 (− 1)	0.2 (0)	37.78	38.67
6	2 (0)	0.1 (− 1)	0.1 (− 1)	36.00	36.44
7	2 (0)	0.2 (0)	0.2 (0)	42.22	41.29
8	2 (0)	0.3 (+ 1)	0.1 (− 1)	40.89	41.33
9	3 (+ 1)	0.3 (+ 1)	0.2 (0)	45.33	44.44
10	1 (− 1)	0.3 (+ 1)	0.2 (0)	41.78	42.67
11	2 (0)	0.2 (0)	0.2 (0)	40.00	41.29
12	3 (+ 1)	0.2 (0)	0.3 (+ 1)	45.33	46.67
13	3 (+ 1)	0.2 (0)	0.1 (− 1)	40.00	40.44
14	1 (− 1)	0.2 (0)	0.3 (+ 1)	44.44	44.00
15	2 (0)	0.1 (− 1)	0.3 (+ 1)	43.11	42.67
16	3 (+ 1)	0.1 (− 1)	0.2 (0)	41.33	40.44
17	2 (0)	0.2 (0)	0.2 (0)	41.33	41.26

For the regression and graphical analysis of experimental data, Statistica 8.0 software (StatSoft, Tulsa, OK, USA) was used. All experiments were performed in three parallels, and the means were taken for analysis.

### Analysis of OTA

In this study, a reaction mixture was used to determine the OTA biodegradation rate of *A. campestris*. The reaction mixture was proposed by Motomura et al. (2003). In the reaction mixture, 500 µL of sodium acetate buffer (0.1 M, pH 5.0) with 485 µL of culture fluid and 15 µL of OTA solution (last concentration, 5 µg/mL) were incubated. The reaction mixture was incubated in the dark at 25 °C for an hour before being stopped with 500 µL of chloroform. After this process, the mixture was centrifuged for 15 min at 1500 rpm, and the lower chloroform layer was evaporated to dryness at room temperature. This process was replicated three times. The OTA-containing residue was separated in 200 µL methanol before being injected into an HPLC (Shimadzu, Japan) to be analyzed for OTA biodegradation (Söylemez et al., 2020).

The sample analysis was performed under the following conditions by HPLC: Macherey–Nagel Nucleodor C18 Column (250 mm × 4 mm, particle size: 5 m); 40 °C; flow rate: 1 mL/min; fluorescent detector with excitation at 312 nm and emission at 333 nm; A standard curve was prepared using various OTA concentrations for calculating the biodegradation rates in all experimental groups. The retention time for OTA by HPLC analysis is presented in Fig. 1.

## Results and discussion

### Biodegradation agent

According to traditional methods, the fruiting body sample belonged to the *Agaricus campestris* species. In addition, the ITS rDNA region sequence of the isolate OBCC 5048 is 99% homologous with existing sequences in the NCBI GenBank with the accession numbers JQ903618.1, HQ446471.1, and JX434655.1, obtained through a BLAST search. Therefore, we can argue that OBCC 5048 is an *Agaricus campestris* isolate. *Agaricus campestris* OBCC 5048's rDNA region sequence was later deposited in GenBank under the accession number MF616403.

### Statistical optimization design

#### Screening of effective factors using Plackett–Burman design

The optimization of cultural conditions is critical for the optimal growth and/or secondary metabolite synthesis in the microbial fermentation process. To select efficient nutritional and/or environmental factors on OTA biodegradation by *Agaricus campestris*, the PB design was used.

The effect of 15 variants during submerged fermentation was investigated. Data were obtained showing that OTA biodegradation occurs with a wide variation (4.9–47.79%). The highest biodegradation was observed in the 3rd trial and the lowest in the 16th trial (Table 1).

**Fig. 2:**
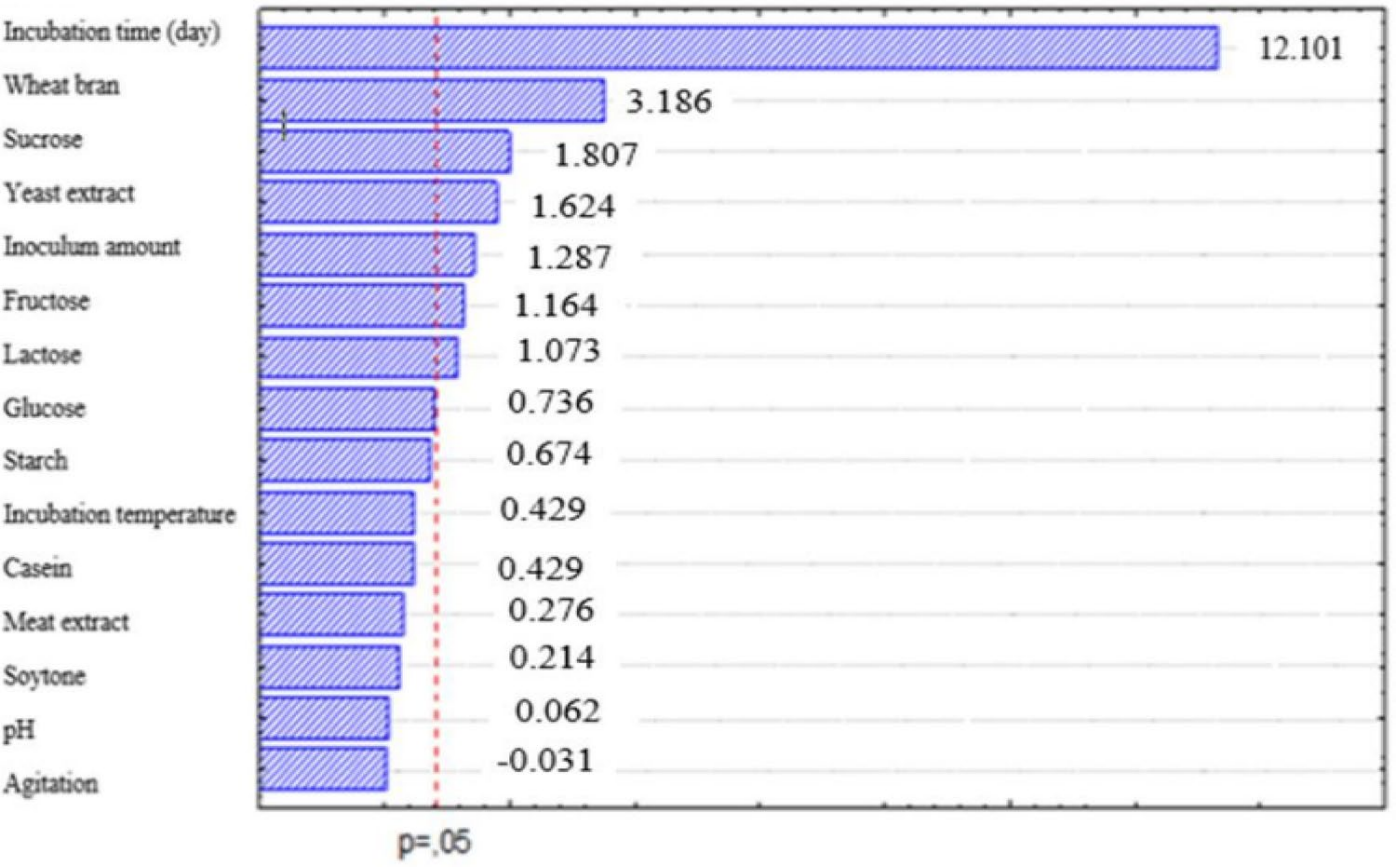
The effect of culture parameters on OTA degradation by Agaricus campestris. E: Effect of variables; R^2^ (coefficient of determination): 0.97551; Adj (adjusted coefficient of determination): 0.96402; Standard error: 6.332954, *Significant at ≤ 0.05

The fundamental effects of the fermentation variants are presented in Fig. 2. The determination coefficient (R^2^) showed the variability of the predicted response compared to the observed one (Fig. 2). The vigorous coefficient of determination value (R^2^ = 0.97551) demonstrates that the data obtained can explain 97.55% of the model and that the model is reliable. According to the analysis results, the regression coefficient showed that all variables except medium pH have an increasing effect on OTA biodegradation.

**Fig. 3:**
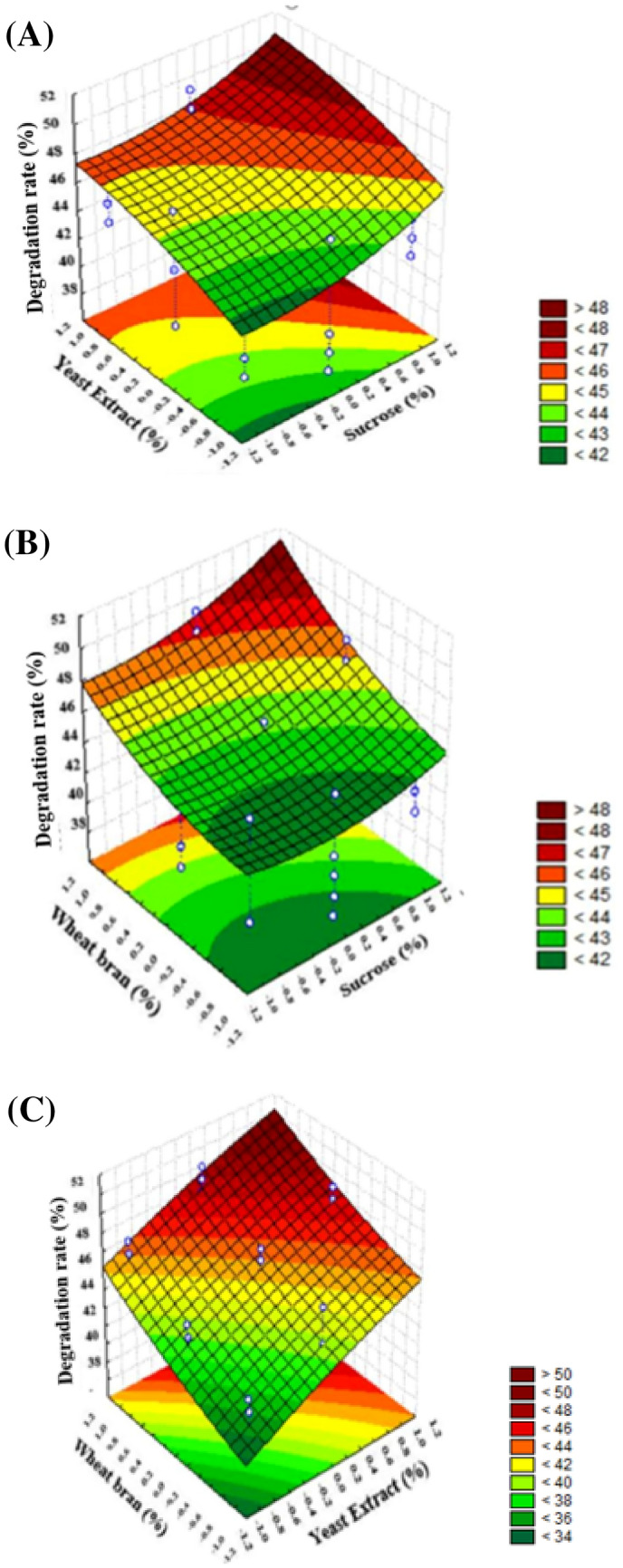
Response surface plots of the experimental variables. (a) sucrose and yeast extract levels (wheat bran 0.3%); (b) sucrose and wheat bran levels (yeast extract 0.3%), and (c) yeast extract and wheat bran levels (sucrose 3%)

Variants tested for OTA biodegradation by *A. campestris* are shown in Fig. 2 as a Pareto chart. These are ordered by their importance. The reference line was used to determine significant variants in this chart and represents a significance level of 0.05. It can be seen apparently from Table 1 D and Fig. 3 (A) that the significant factors for OTA biodegradation by *A. campestris* were sucrose (p: 0.000021), fructose (p: 0.003056), lactose (0.005860), wheat bran (p: 0.000000), yeast extract (p: 0.000092), incubation time (p: 0.000000), and inoculum amount (p: 0.001238). Sucrose (X_3_) and yeast extract (X_6_) have the highest effects on OTA biodegradation among the tested carbon (X_1_–X_5_) and nitrogen (X_6_–X_9_) sources (Fig. 2). Owing to their importance, the inoculum amount (X_14_) and incubation time (X_15_) were chosen as stationary fermentation ingredients for the following studies. Consequently, sucrose (X_3_), yeast extract (X_6_), and wheat bran (X_10_) were chosen for further studies to figure out the impact of their interaction on OTA biodegradation by *A. campestris*.

#### Optimization of significant variables using Box–Behnken methodology

Following the PB design, the Box–Behnken methodology was used to investigate the optimization of the selected medium ingredients. The predicted and experimental OTA biodegradation results and the design matrix of the total of 17 trials are demonstrated in Table 2. The control trial (central values) was studied five times.

The maximum experimental value for OTA biodegradation were 46.67% in 12th run. The quadratic regression model's results of the variance analysis are represented in Table 2. The definition coefficient for OTA biodegradation was calculated at 0.74. It means that there is a good correlation between variables and OTA biodegradation, and 74% of the differences in the response can be explained by the Box–Behnken methodology.

By calculating the regression coefficient, a quadratic polynomial equation was obtained:

$${\text{Degradation of the OTA}}\left( \% \right) = {41},{285} + 0.{89}0{\text{ X}}_{{3}} + 0.{\text{689 X}}_{{3}}^{{2}} + {2}.000{\text{ X}}_{{6}} {-}0.{42}0{\text{ X}}_{{6}}^{{2}} + {2}.{\text{667X}}_{{{1}0}} + \, 0.{\text{691X}}_{{{1}0}}^{{2}} + 0.00{\text{ X}}_{{3}} {\text{X}}_{{6}} + 0.{\text{444 X}}_{{3}} {\text{X}}_{{{1}0}} {-}0.{\text{445 X}}_{{6}} {\text{X}}_{{{1}0}}$$

where the predicted response is OTA biodegradation (%); sucrose, yeast extract, and wheat bran are coded as X_3_, X_6_, and X_10_, respectively. We can clearly see from the formula that these three variables have positive effects on OTA biodegradation. The performed model’s regression analysis shows high significance thanks to the F-test’s low p value (< 0.01).

While the third is kept at constant levels, the mutual effects of the two variables are shown in Fig. 3. We can argue that, among other carbon sources, sucrose increases the expression of the enzyme that causes OTA degradation by *A. campestris*. Wheat bran interacted positively with yeast extract. In addition to that, sucrose also interacted positively with wheat bran and yeast extract according to OTA biodegradation. As a result of the analysis of the OTA biodegradation data of the *A. campetris* isolate obtained by the response surface test design, the optimal conditions for biodegradation were; sucrose 3%, yeast extract 0.3%, and wheat bran 0.3%, soytone 0.5%, KH_2_PO_4_ 0.2%, MgSO_4_⋅7H_2_O 0.05%, CaCl_2_⋅H_2_O 0.01%, ammonium tartrate 0.2%, trace element solution 1%; 28 °C of incubation temperature, medium pH 5, 7.5 inoculum amount, 125 rpm of agitation speed, and 12 days of incubation period. The maximum biodegradation rate under optimum conditions was 46.67%. The model was validated by repeating the experiments under optimized conditions that resulted in 41.19% OTA biodegradation and the validity of the model was verified. On the other hand, this ratio was 24.94% under non-optimized conditions.

To our knowledge, the use of statistical experimental designs for the optimization of OTA biodegradation has rarely been seen in the literature previously (Rahmani et al., 2011). The use of statistical designs has some advantages such as reliable metabolite/enzyme production, low-cost and simplicity. The type and concentration of carbon and nitrogen sources, as well as the C/N ratio, are critical for microorganisms to produce enzymes and metabolites. In this study, yeast extract and sucrose were determined to be the best nitrogen and carbon sources, among others. Sucrose is rarely used for OTA biodegradation as a carbon source by fungi. Xiong et al. (2021) screened four different growth media to optimize OTA biodegradation by *A. oryzae*. Among the media used, *A. oryzae* gave the highest OTA biodegradation rate (94%) in media containing yeast extract and sucrose over a 72-h period (Xiong et al., 2021). On the other hand, yeast extract as a nitrogen source has been used by microorganisms such as *Aspergillus niger* and *Eubacterium biforme* to OTA biodegradation (Zhao et al., 2020).

Some of the studies have shown that the cell wall components of microorganisms used in OTA detoxification and different culture conditions have effects on OTA absorption. Although adsorption causes a decrease in OTA, it does not have a high biodegradation rate and permanent detoxification like crude or purified enzymes (Chen et al., 2018). Some researchers studied the biodegradation of OTA by pure enzymes. Several commercial proteases, for example, can hydrolyze ochratoxin A into ochratoxin α in varying amounts. After an incubation period of 25 h, significant hydrolytic activity at pH 7.5 for protease A (87.3%) and for pancreatin (43.4%) was detected (Abrunhosa et al., 2010). In another study, 23 lipases and esterases were screened for their ability to hydrolyze OTA. When the results were analyzed quantitatively, they revealed that only a lipase preparation from *A. niger* (Amano A) was able to degrade OTA. The specific activity of the crude lipase was determined to be 7.63 units/µg (Stander et al., 2000). In the study in which the biodegradation capacity of a fusion enzyme with zearalenone and OTA was investigated, 100% biodegradation of OTA was realized at pH 7.0, 30 °C, and 30 min. Recombinant DNA was used to create the fusion enzyme zearalenone hydrolase-carboxypeptidase (ZHDPC), which combines the genes for zearalenone hydrolase (ZHD) and carboxypeptidase (CP).

OTA enzymatic biodetoxification was reported only four years after its discovery (Pitout, 1969). Thenceforward, a lot of microorganisms were studied by researchers for their biodegradation capacity. However, *Agaricus campestris* is optimized for OTA biodegradation for the first time in this study. The aim of this study is to determine the best conditions for OTA biodegradation and use them for producing enzymes using recombinant DNA techniques in the future. Chang et al. (2014) studied *Bacillus amyloliquefaciens,* and they determined carboxypeptidase as the responsible enzyme. They compared co-cultivation with the supernatant of the crude enzyme (OTA producer and *Bacillus amyloliquefaciens* ASAG1) and the purified protein of carboxypeptidase from *Bacillus amyloliquefaciens* ASAG1. As a result, OTA decreased by 41% and 72% when co-cultivated with the supernatant of the crude enzyme and the purified protein of carboxypeptidase, respectively (Chang et al., 2014). There is another study on *Bacillus spp.* and *B. subtilis* CW14, which degraded 47.10% of OTA (Shi et al., 2014). In the other study, the dacA and dacB genes responsible for carboxypeptidase production from the *B. subtilis CW* 14 strain were expressed in *E. coli*. The purified dacA protein provided 71.3% OTA biodegradation in 24 h (Xu et al., 2021). Ochratoxinase (OTase) obtained from *A. niger* strain W-35 was successfully expressed in *E. coli* BL21 and degraded OTA at a ratio of 85.1% for 12 h (Zhao et al., 2020). *Eubacterium biforme* was isolated from the swine intestinal system, and this bacterium detoxified more than 75% of OTA in liquid media (Upadhaya et al., 2012). Lactic acid bacteria, such as *Pediococcus parvulus,* have also been determined to be successful in OTA biodegradation (Muhialdin et al., 2020). Engelhardt (2002) reported OTA biodegradation by macrofungi for the first time with *P. chrysosporium* and *P. ostreatus* (Engelhardt, 2002).

Box–Behnken’s response surface was used as an experimental design for evaluating the effect of three factors on the percentage of reduced OTA interest. In this study, 17 trials with 3 variables were used. Trial number 12 had the best OTA biodegradation percentage of the three variables studied. Expected and observed results in all trials are in agreement.

There are some OTA biodegradation studies that have been performed with reaction mixtures containing various organisms and their culture fluids (Table 3). In these studies, the biodegradation rates ranged from 41 to 100%. However, considering the standard amount of OTA in the reaction mixture and reaction times, our study has important advantages compared to other studies. *Trichosporon mycotoxinivorans* is a well-known OTA and zearalenone-degrading yeast. However, the maximum OTA biodegradation could be obtained after 5 h of incubation time with this yeast. In other reports for OTA biodegradation, the incubation time has been applied as 24 h, 48 h, or even 5–16 days (Petchkongkaew et al., 2008; Varga et al., 2005). On the other hand, a biodegradation of around half of OTA occurred in a short period of 1 h in this study. This rate is even better than the 24-h incubation of *S. cerevisiae*. As a result, *Agaricus campestris* appears to have better potential under these conditions than most other organisms.

**Table 3:** Some of OTA degrading microorganisms and degradation conditions

Microorganism		Reaction mixture (Culture Fluid: Toxin:Buffer)	Reaction time	Toxin concentration (mg/L)	Degradation ratio (%)	References
Bacteria	*Bacillus licheniformis*	Culture fluid	48 h	5.0	92.5	Petchkongkaew et al. (2008)
	*L. bulgaricus*	Culture fluid	24 h	0.05	94	Böhm et al. (2000)
	*L. acidophilus*	Culture fluid	4 h	0.5—1	≥ 95	Fuchs et al. (2008)
	*B. licheniformis*	Culture fluid	24 h	0.05	68	Böhm et al. (2000)
	*L. helveticus*	Culture fluid	24 h	0.05	72	Böhm et al. (2000)
Yeast	*Trichosporon mycotoxinivorans*	Culture fluid	5 h	0.2	100	Molnar et al. (2004)
	*Phaffia rhodozyma*	Culture fluid	24 h	7.5	90	Péteri et al. (2007)
	*Saccharomyces cerevisiae*	Culture fluid	24 h	0.3	41	Pitrowska and Zakowska (2000)
Filamentous Fungi	*Rhizopus homothallicus*	Culture fluid	16 days	7.5	≥ 95	Varga et al. (2005)
	*R. oryzae*	Culture fluid	16 days	7.5	≥ 95	Varga et al. (2005)
	*R. stolonifer*	Culture fluid	16 days	7.5	≥ 95	Varga et al. (2005)
	*Aspergillus carbonarius*	Culture fluid	5 days	2.0	≥ 80	Bejaoui et al. (2006)
	*A. niger*	Culture fluid	5 days	2.0	≥ 80	Bejaoui et al. (2006)
	*A. japonicus*	Culture fluid	5 days	2.0	≥ 80	Bejaoui et al. (2006)
	*Pleurotus ostreatus*	Culture fluid	5 days	2.0	77	Engelhardt (2002)
	*Agaricus campestris*	445:15:500	1 h	5.0	47	This Study

Two metabolic biodegradation pathways are known for OTA (Karlovsky, 1999). Although the OTA biodegradation products have not been reported in this study, the toxicity after biodegradation has been presented in another report (Söylemez and Yamaç, 2021). In this report, the toxicity was decreased by 46.20%.

In conclusion, the optimum nutritional conditions for maximum OTA biodegradation by *Agaricus campestris* were (g/L) yeast extract 3, sucrose 30, soytone 5, wheat bran 3, ammonium tartrate 2, CaCl_2_⋅H_2_O 0.1, KH_2_PO_4_ 2, MgSO_4_⋅7H_2_O 0.5, and trace element solution 10 mL/L. Other optimal conditions included a medium pH of 5.0, an incubation temperature of 28 °C, an agitation speed of 125 rpm, an incubation period of 12 days, and an inoculum amount of 7.5%. According to other published reports, it can be argued that *Agaricus campestris* has a high capacity based on its OTA biodegradation ratio in just 1 h of reaction time. Under the optimum conditions, the rate of OTA biodegradation was determined as 46.67% after an hour of incubation. To the best of our knowledge, this is the first report on the optimization of OTA biodegradation by *Agaricus campestris*. Our next report will focus on the determination, purification, and characterization of the responsible enzyme of *Agaricus campestris* for OTA biodegradation, which has not yet been reported.
